# Therapeutic Implications of Ceritinib in Cholangiocarcinoma beyond ALK Expression and Mutation

**DOI:** 10.3390/ph17020197

**Published:** 2024-02-02

**Authors:** Kyaw Zwar Myint, Brinda Balasubramanian, Simran Venkatraman, Suchada Phimsen, Supisara Sripramote, Jeranan Jantra, Chaiwat Choeiphuk, Somkit Mingphruedhi, Paramin Muangkaew, Narongsak Rungsakulkij, Pongsatorn Tangtawee, Wikran Suragul, Watoo Vassanasiri Farquharson, Kanokpan Wongprasert, Somchai Chutipongtanate, Pimtip Sanvarinda, Marisa Ponpuak, Naravat Poungvarin, Tavan Janvilisri, Tuangporn Suthiphongchai, Kiren Yacqub-Usman, Anna M. Grabowska, David O. Bates, Rutaiwan Tohtong

**Affiliations:** 1 Graduate Program in Molecular Medicine, Faculty of Science, Mahidol University, Bangkok 10400, Thailand; kyawzwar.myn@student.mahidol.ac.th (K.Z.M.); brinda.balasubramanian@nottingham.ac.uk (B.B.); simran.ven@mahidol.ac.th (S.V.); tavan.jan@mahidol.ac.th (T.J.); 2 Translational Medical Sciences Unit, School of Medicine, University of Nottingham, Nottingham NG7 2RD, UK; 3 Department of Biochemistry, Faculty of Medical Science, Naresuan University, Phitsanulok 65000, Thailand; suchadaph@nu.ac.th (S.P.); chaiwatch1996@gmail.com (C.C.); 4 Department of Biochemistry, Faculty of Science, Mahidol University, Bangkok 10400, Thailand; tangmo5405243@gmail.com (S.S.); jantrabukae@gmail.com (J.J.); tuangporn.sut@mahidol.ac.th (T.S.); 5 Hepato-Pancreatic-Biliary Surgery Unit, Department of Surgery, Faculty of Medicine, Ramathibodi Hospital, Mahidol University, Bangkok 10400, Thailand; somkit.min@mahidol.ac.th (S.M.); paramin.mua@mahidol.ac.th (P.M.); narongsak.sun@mahidol.ac.th (N.R.); pongsatorn.tan@mahidol.ac.th (P.T.); wikran.sur@mahidol.ac.th (W.S.); watoo.vas@mahidol.ac.th (W.V.F.); 6 Department of Anatomy, Faculty of Science, Mahidol University, Bangkok 10400, Thailand; kanokpan.won@mahidol.ac.th; 7 Division of Epidemiology, Department of Environmental and Public Health Sciences, University of Cincinnati College of Medicine, Cincinnati, OH 45267, USA; 8 Department of Pharmacology, Faculty of Science, Mahidol University, Bangkok 10400, Thailand; pimtip.san@mahidol.ac.th; 9 Department of Microbiology, Faculty of Science, Mahidol University, Bangkok 10400, Thailand; marisa.pon@mahidol.ac.th; 10 Department of Clinical Pathology, Faculty of Medicine, Siriraj Hospital, Mahidol University, Bangkok 10700, Thailand; naravat@gmail.com; 11 Biodiscovery Institute, University of Nottingham, Nottingham NG7 2RD, UK; kiren_yacqub@hotmail.co.uk (K.Y.-U.); anna.grabowska@nottingham.ac.uk (A.M.G.); david.bates@nottingham.ac.uk (D.O.B.)

**Keywords:** cholangiocarcinoma, ceritinib, cancer, kinase inhibitors, antitumor agents

## Abstract

Cholangiocarcinoma (CCA) is a difficult-to-treat cancer, with limited therapeutic options and surgery being the only curative treatment. Standard chemotherapy involves gemcitabine-based therapies combined with cisplatin, oxaliplatin, capecitabine, or 5-FU with a dismal prognosis for most patients. Receptor tyrosine kinases (RTKs) are aberrantly expressed in CCAs encompassing potential therapeutic opportunity. Hence, 112 RTK inhibitors were screened in KKU-M213 cells, and ceritinib, an approved targeted therapy for ALK-fusion gene driven cancers, was the most potent candidate. Ceritinib’s cytotoxicity in CCA was assessed using MTT and clonogenic assays, along with immunofluorescence, western blot, and qRT-PCR techniques to analyze gene expression and signaling changes. Furthermore, the drug interaction relationship between ceritinib and cisplatin was determined using a ZIP synergy score. Additionally, spheroid and xenograft models were employed to investigate the efficacy of ceritinib in vivo. Our study revealed that ceritinib effectively killed CCA cells at clinically relevant plasma concentrations, irrespective of ALK expression or mutation status. Ceritinib modulated multiple signaling pathways leading to the inhibition of the PI3K/Akt/mTOR pathway and activated both apoptosis and autophagy. Additionally, ceritinib and cisplatin synergistically reduced CCA cell viability. Our data show ceritinib as an effective treatment of CCA, which could be potentially explored in the other cancer types without ALK mutations.

## 1. Introduction

Cholangiocarcinoma (CCA) or the cancer of the bile ducts is the second most common primary hepatic malignancy after hepatocellular carcinoma, constituting 15% of all primary liver tumors and 3% of gastrointestinal malignancies [1,2,3]. The incidence and mortality rates of CCA are increasing around the world [4,5]. Thailand bears the highest incidence of CCA globally, with a mortality rate of 14% leading to 20,000 CCA-related deaths annually, thereby posing a significant burden on the nation [6,7]. CCA is usually diagnosed in advanced stages with a dismal prognosis and a discouraging 7–20% 5-year survival rate [8,9,10]. Treatment options for CCA are limited, with surgical resection and liver transplant being the only forms of treatment with curative intent, but both are technically challenging and restricted to patients diagnosed in the early stages. CCA patients in advanced stages are generally inoperable, restricting the treatment options for many patients to solely palliative chemotherapy [8,11]. Moreover, current diagnostic methods are incapable of detecting tumors at earlier stages, thereby leading to a growing number of patients diagnosed at more advanced stages when the cancer has already metastasized [9]. 

Despite efforts to employ adjuvant or neoadjuvant chemotherapy following surgical resection to reduce local recurrence, no clear improvement in survival was seen [12]. Moreover, patients diagnosed at later stages with progressive disease have limited options and are generally restricted to palliative chemotherapy [8,11,13]. Furthermore, many patients may not be healthy enough to receive aggressive systemic therapy. Despite some patients benefiting from the current standard of care, others fail to respond to first-line chemotherapy, likely due to the aggressive and diverse nature of CCA [14]. Therefore, there is a pressing need for effective treatments to combat aggressive CCA tumors.

CCAs exhibit significant overexpression and/or mutations in receptor tyrosine kinases (RTKs), including epidermal growth factor receptors (EGFRs), fibroblast growth factor receptors (FGFRs), vascular endothelial growth factor receptors (VEGFRs), and various abnormalities in signaling pathways such as Interleukin-6 receptor, receptor tyrosine kinases, PI3K/PTEN/AKT/mTOR, and KRAS/BRAF/MEK/ERK [15]. These aberrations present promising targets for targeted therapies.

Receptor tyrosine kinase inhibitors (RTKis) are specifically designed to impede the kinases responsible for phosphorylating downstream targets [16]. Using the KKU-M213 cell line, we conducted a screening of 112 RTK inhibitors (RTKis), and ceritinib emerged as the most cytotoxic compound (Supplementary Data Table S1). Ceritinib, marketed as Zykadia^®^ by Novartis, is an approved targeted therapy for anaplastic lymphoma kinase (ALK)-positive non-small cell lung cancer (NSCLC) patients. It is employed as a first- or second-line treatment either as a monotherapy or in combination with other therapeutic agents [17,18]. The primary mode of action of ceritinib in ALK-positive NSCLC patients involves the inhibition of the driver mutation, characterized by the fusion of the 3′ segment of the ALK kinase region with the 5′ portion of another gene, resulting in a constitutively active kinase that triggers downstream signaling pathways [19]. Ceritinib has demonstrated antitumor activity in hepatocellular carcinoma cells by inhibiting the ALK and insulin-like growth factor 1 receptor (IGF1R) signaling pathways [20]. Additionally, it has been shown to sensitize glioblastoma cells to temozolomide, a chemotherapy drug employed in the treatment of brain tumors [21]. In vitro and in vivo studies have established that ceritinib inhibits cell proliferation, migration, and invasion, while also inducing apoptosis in pancreatic cancer [22].

Recently, we have reported ceritinib as a potential therapeutic agent in CCA even in the absence of aberrant ALK/ROS1 (ROS Proto-Oncogene 1) expression [23]. The serendipitous finding of ceritinib as one of the most cytotoxic compounds to KKU-M213 in RTKis’ screening led us to further our study of ceritinib to clarify and reaffirm its cytotoxicity, ALK expression and mutation status, the mechanisms of cell death, and the associated signaling pathways in CCA.

Our results substantiated that, without ALK rearrangement or mutations, ceritinib is particularly cytotoxic to CCA cells compared to non-CCA cells. Ceritinib efficiently induces cell death in CCA cells through the activation of both apoptosis and autophagy pathways, irrespective of ALK mutation or expression status by targeting multiple kinases leading to the inhibition of the Akt signaling pathway. A comprehensive drug-synergy study showed ceritinib, at low concentration, synergistically killed CCA cells with cisplatin, and at high concentration it is cytotoxic as a single agent. The observation in spheroid models and xenograft models encompasses the potential clinical applicability of ceritinib in CCA.

## 2. Results

### 2.1. Receptor Tyrosine Kinase Inhibitor Screening in CCA

**Figure 1:**
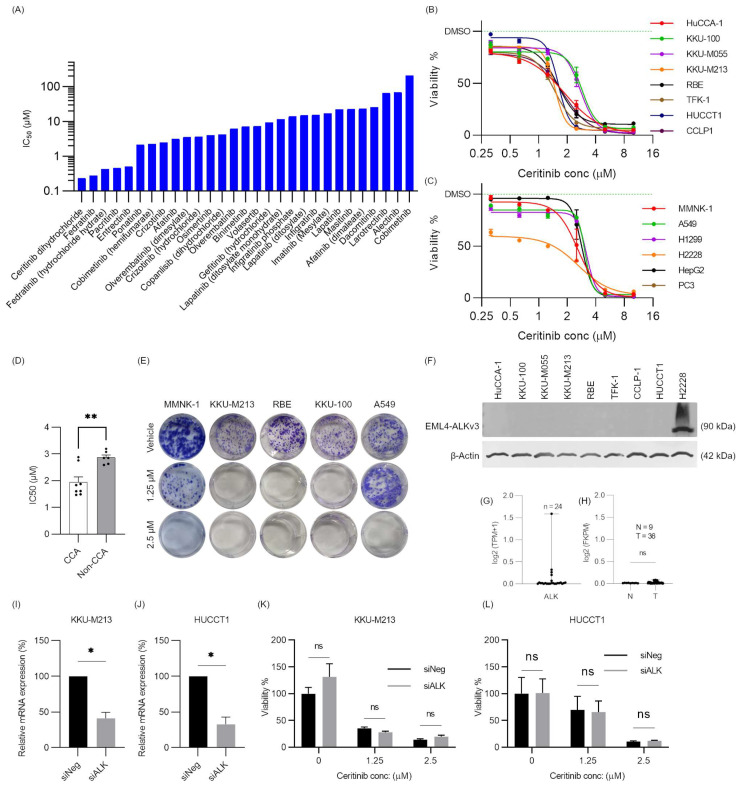
Ceritinib is particularly cytotoxic to CCA cells and cytotoxic mechanism is ALK-expression/mutation independent. (**A**) LogIC_50_ of secondary screening of RTKis, (**B**) viability of CCA cell lines, and (**C**) non-CCA cell lines at 72 h after ceritinib treatment (0.3125, 0.625, 1.25, 2.5, 5, and 10 μM). (**D**) Comparison of IC_50_s of CCA vs. non-CCA cell lines and (**E**) clonogenic assay of immortalized cholangiocytes (MMNK-1), CCA (KKU-M213 and RBE, KKU-100), and non-CCA (A549) cell lines at 10 days after ceritinib treatment. (**F**) Western blot of ALK expression in CCA cell lines. H2228 is EML4-ALKv3 fusion expressing positive control cell line. ALK mRNA expression in (**G**) CCA cell lines of CCLE database and (**H**) tumor adjacent normal (N = 9) vs. tumor (T = 36) of TCGA database. qRT-PCR analysis of siRNA-mediated gene silencing of ALK expression in (**I**) KKU-M213 and (**J**) HUCCT1 cell lines. Viability of (**K**) KKU-M213 and (**L**) HUCCT1 cells after ALK silencing with/without ceritinib (1.25 or 2.5 μM) treatment. The data are presented as means ± SEM of three independent experiments performed with triplicate wells. * *p* < 0.005 and ** *p* < 0.01 compared to vehicle or negative control.

Primary screening of 112 RTKis in KKU-M213 cells (Table S1) showed 29 RTKis had potent cytotoxicity to KKU-M213 cells at 10 μM. Secondary screening (0.01–10 μM) found ceritinib, fedratinib, pacratinib, and entrectinib had IC_50_s of less than 1 μM (Table S2, Figure S1). Considering ceritinib was the most cytotoxic compound, its efficacy in CCA was further investigated (Figure 1A). The conventional mechanism of action of ceritinib, to inhibit phosphorylation of ALK, was proven in H2228, echinoderm microtubule-associated protein-like 4-anaplastic lymphoma kinase version 3 (EML4-ALKv3) positive cell line (Figure S2).

### 2.2. Ceritinib Is Particularly Cytotoxic to CCA Cells

CCA cell lines (HuCCA-1, KKU-100, KKU-M055, KKU-M213, RBE, TFK-1, HUCCT1, and CCLP1), along with an immortalized cholangiocyte cell line (MMNK-1), non-small cell lung cancer cell lines (NSCLC) harboring normal ALK gene (A549 and H1299), an EML4-ALKv3 mutation positive cell line (H2228), hepatoblastoma cell line (HepG2), and a prostate cancer cell line (PC3), were subjected to ceritinib treatment for 72 h at concentrations ranging from 0.625 to 10 μM. Two-fold dilutions of ceritinib concentrations were used to observe dose–response curve changes. All CCA cell lines and HepG2 (hepatoblastoma cell line) exhibited sensitivity to ceritinib, with the IC_50_s falling within or below the range of clinical plasma concentrations (1.4–2.3 µM) [24,25]. In the immortalized cholangiocyte cell line (MMNK-1), the IC_50_ was determined to be 2.49 ± 0.16 μM. A549 (IC_50_ = 2.63 ± 0.93 μM), H1299 (IC_50_ = 2.44 ± 0.27 μM), and PC3 (IC_50_ = 2.96 ± 0.46 μM) demonstrated relatively lower sensitivity to ceritinib compared to CCA cells. H2228 exhibited the highest sensitivity to ceritinib, with an IC_50_ of 0.84 ± 0.09 µM (Figure 1B,C). Interestingly, IC_50_s of CCA cells are significantly lower than those of non-CCA cells (*p* = 0.0028) (Figure 1D). Clonogenic assay confirmed that ceritinib was more cytotoxic to KKU-M213, RBE and KKU-100 cell lines (CCA) compared to MMNK-1 (immortalized cholangiocyte) and A549 cells (non-CCA) at 1.25 μM (Figure 1E). 

### 2.3. Ceritinib-Induced Cytotoxicity in CCA Cells Is ALK Mutation/Expression Independent

Ceritinib is a potent second-generation tyrosine kinase inhibitor with high ALK selectivity [26]. However, western blot (WB) analysis showed there was no detectable protein expression of ALK standard forms or fusion protein in CCA cell lines, in comparison to the EML4-ALKv3 fusion protein positive H2228 cell line (Figure 1F). RNA sequencing data from the Cancer Cell Line Encyclopedia (CCLE) database revealed low transcript abundance of ALK in CCA cell lines (log2TPM + 1 < 0.5) (Figure 1G) [27] and analysis of “The Cancer Genome Atlas” (TCGA) dataset revealed no significant difference in ALK mRNA expression between normal bile duct (tumor adjacent normal) (N = 9) and CCA tumor (T = 36), and both exhibited notably low expressions (log2 (FKPM) < 0.5) (Figure 1H). This finding was further supported by qRT-PCR, which showed high Ct values (>35) in KKU-M213 and HUCCT1, and no Ct value for ALK mRNAs in HuCCA-1, KKU-M055, KKU-100, RBE, and TFK-1 compared to the internal control 18S rRNA (Ct values of 10 to 13). Moreover, we confirmed in the CCLE database that these cell lines do not harbor ALK mutations or fusions [27]. 

Not only is it the case that CCA cells lacking ALK fusions exhibited sensitivity to ceritinib, but also siRNA-mediated gene silencing in KKU-M213 and HUCCT1 cells did not impact cell viability or alter the cytotoxic effects of ceritinib when compared to cells treated with negative siRNA control (Figure 1I–L). Moreover, ceritinib is more cytotoxic to the CCA cells than the other ALK inhibitors (Alectinib, ASP3026 and NVP-TAE684) (Figure S3A–D). These findings collectively indicated that ceritinib exerts a unique cytotoxic mechanism in CCA cells independent of ALK expression or mutation status.

### 2.4. Prediction of Ceritinib-Induced Cell Death Mechanism and Signaling Pathways

To understand the cytotoxic mechanisms of ceritinib in CCA, a literature search, datamining, and analysis were performed. KINOMEscan^®^ (in vitro) data from the Harvard Medical School LINCS (Library of Integrated Network-Based Cellular Signatures) project revealed that ceritinib can effectively inhibit multiple kinases at a concentration of 1 μM [28,29]. Figure S4A illustrates that 57 kinases can be inhibited by up to 50% by ceritinib. Mutated forms of these kinases are not expressed in the CCA cell lines according to the CCLE database [27]. Among them, although IGF1R and FAK1 are reported as non-canonical targets of ceritinib [30,31,32], GSK1904529A (an IGF1R and InsR inhibitor) or FAK inhibitor 14 did not induce cytotoxic effects in CCA cells at the same concentration as ceritinib (Figure S4B). Given the promiscuity of ceritinib in kinase inhibition and potential heterogenous expression of these kinases in an individual cell line/patient, we hypothesized that the signaling changes affected by the inhibition of multiple kinases may be the keys to cell death in CCA. As such, KEGG (Kyoto Encyclopedia of Genes and Genomes) pathway enrichment analysis was performed to identify the molecular interaction, reaction, and relation network where these 57 kinases are particularly abundant and our analysis showed that MAPK and mTOR signaling pathways and autophagy appeared to be the top candidate pathways and mechanisms (Figure S4C).

### 2.5. Ceritinib-Induced Apoptosis and Autophagy in CCA

**Figure 2:**
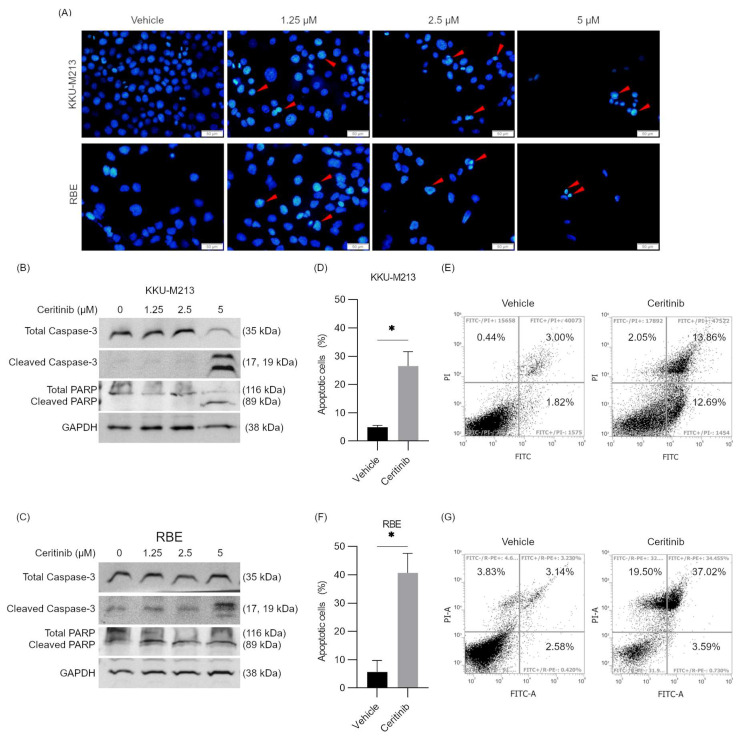
Ceritinib-induced apoptosis in CCA cells. (**A**) DAPI staining shows ceritinib treatment for 24 h induced chromatin condensations and nuclear fragmentations (red arrows) in KKU-M213 and RBE cells. Western blot of caspase-3 and PARP cleavage after ceritinib (0, 1.25, 2.5, and 5 μM) treatment for 24 h in (**B**) KKU-M213 and (**C**) RBE cells. Apoptotic cells (%) and representative scattered plots of FACS in (**D**,**E**) KKU-M213 and (**F**,**G**) RBE cells after 5 μM ceritinib treatment for 24 h. The data are presented as means ± SEM of three independent experiments. * *p* < 0.05 compared to vehicle.

To further understand ceritinib-induced cytotoxicity in CCA cells, the mechanism of cell death was explored. DAPI (4′,6-diamidino-2-phenylindole) staining at 24 h after ceritinib treatment showed a dose-dependent decrease in cell numbers, chromatin condensations, and nuclear fragmentations, which are the characteristics of apoptotic cells (Figure 2A). Additionally, western blot analysis demonstrated the cleavage of caspase-3, along with a decrease in total caspase-3 levels, and the subsequent cleavage of poly (ADP-ribose) polymerase 1 (PARP) in KKU-M213 and RBE cells following 24 h of ceritinib treatment (Figure 2B,C). Fluorescence-activated cell sorting (FACS) analysis with annexin V and PI staining further confirmed the induction of apoptosis, as treatment with 5 µM ceritinib for 24 h significantly increased the population of apoptotic cells (annexin V+) in KKU-M213 (from 4.82 ± 0.74% to 26.55 ± 5.04% and *p* = 0.045) and RBE (from 5.72% to 40.61% and *p* = 0.011) cells (Figure 2D–G). These findings collectively demonstrate that ceritinib induces apoptotic cell death in CCA cells, as evidenced by the morphological changes, caspase-3 activation, and increased annexin V positivity.

**Figure 3:**
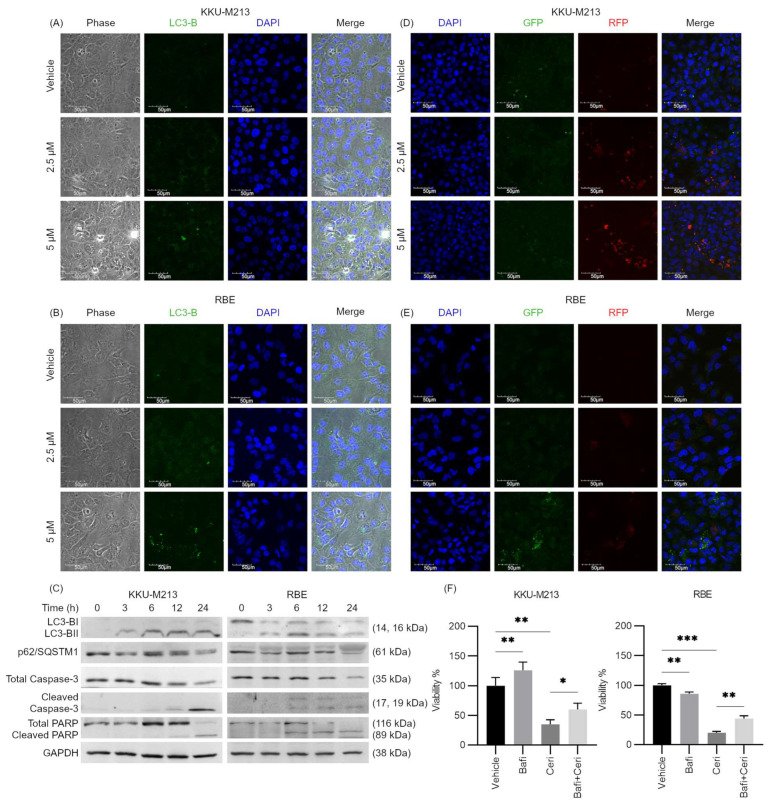
Ceritinib-induced autophagy in CCA cells. Vacuolations in (**A**) KKU-M213 and (**B**) RBE cells, which coincide with the LC3B expression (green) at 6 h after ceritinib treatment. (**C**) Western blot analysis shows time-dependent increase in LC3-BII formation and decrease in p62 expression followed by caspase 3 cleavage and PARP cleavage in KKU-M213 and RBE cells. (**D**,**E**) Ceritinib treatment increased the RFP/GFP ratio in GFP-RFP-LC3B transfected KKU-M213 and RBE cells. (**F**) 100 nM Bafilomycin A1 rescued the ceritinib-induced cytotoxicity in KKU-M213 and RBE cells. * *p* < 0.005, ** *p* < 0.01, and *** *p* < 0.001 compared to vehicle.

Interestingly, within 3–6 h of ceritinib treatment, we observed numerous vacuolations in the cytoplasm of the cells. Figure 3A,B depicts the vacuolations observed in KKU-M213 and RBE cells after 6 h of ceritinib treatment. Immunofluorescence staining with LC3-B (microtubule-associated protein 1 light chain 3 beta), a marker of the autophagosome, revealed its co-localization with these vacuoles, while DAPI staining indicated the absence of nuclear fragmentations or shrinkage at this time point. Time dependent western blot analysis demonstrated an increase in LC3-BII formation as early as 3 h after treatment, followed by caspase-3 cleavage at 6 h, PARP cleavage at 6–24 h, and a reduction in sequestosome 1 (p62/SQSTM1) levels at 24 h (Figure 3C). These findings suggest that ceritinib treatment triggers autophagy as indicated by the formation of autophagosomes in the early hours, which is subsequently followed by apoptosis at later time points.

To determine whether the observed increase in LC3-BII was due to autophagy flux inhibition, GFP-LC3-RFP plasmid was transfected into KKU-M213 and RBE cells. Following ceritinib treatment, an elevation in the RFP/GFP ratio was observed (Figure 3D,E), suggesting that the vacuoles had fused with the lysosome, leading to quenching of GFP. Hence, our data suggest that the increase in LC3-BII is likely due to autophagy induction, but not autophagy flux inhibition.

Notably, autophagy may serve as a mechanism of drug resistance, a survival response to ceritinib treatment [33]. To determine if this was the case, we inhibited autophagy using bafilomycin A1 (Baf1) and observed that ceritinib-induced cytotoxicity was reversed in both KKU-M213 (*p* = 0.0224) and RBE (*p* = 0.0068) cells. These results indicate that ceritinib-induced autophagy is not a survival but rather a cell death pathway in these cells (Figure 3F).

### 2.6. Ceritinib Alters Multiple Signaling Pathways of CCA Cells

**Figure 4:**
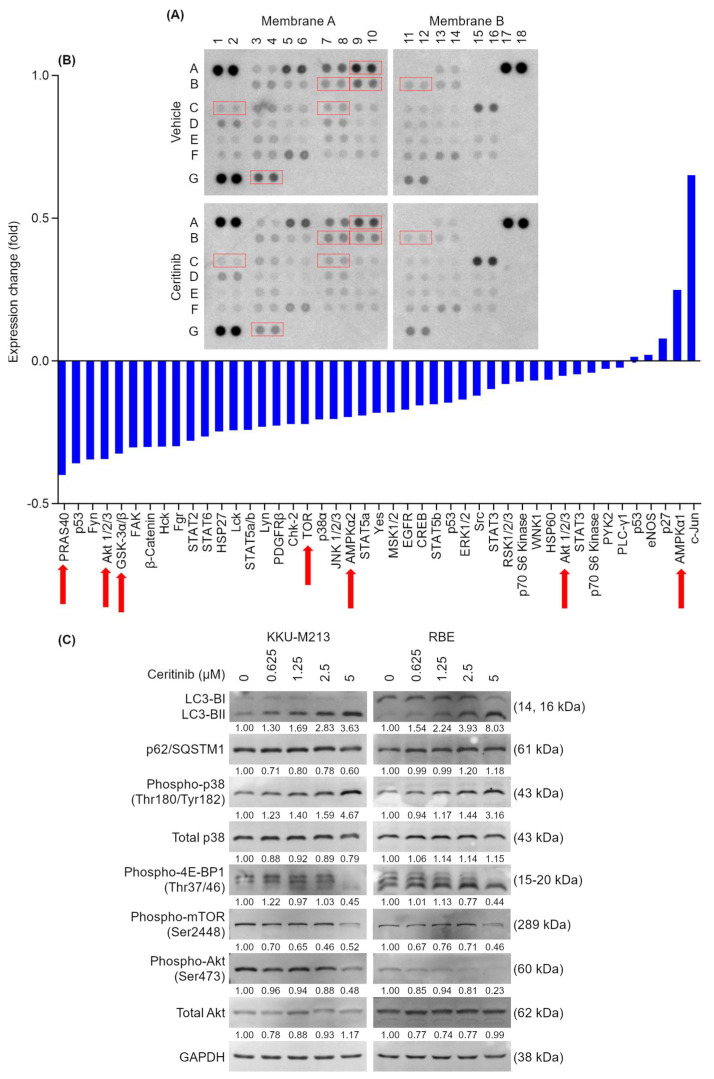
Signaling changes induced by ceritinib treatment in KKU-M213 cells. KKU-M213 cells were treated with ceritinib for 6 h. Total protein extraction was performed for antibody array analysis. (**A**) The array blots of KKU-M213 cells treated with vehicle or ceritinib and (**B**) relative fold change in phospho-proteins. Red arrows and boxes indicate the proteins associated with Akt signaling pathway (**C**) Western blot of LC3-B, p62/SQSTM1, phospho-p38, total p38, phospho-4E-BP1, phospho-mTOR, phospho-Akt, and total Akt changes at 6 h after ceritinib treatment (0.625, 1.25, 2.5, and 5 μM). Numbers indicate the average of densitometry analysis of four independent experiments.

To identify the signaling pathways involved in ceritinib-induced cytotoxicity in CCA cell lines, a Proteome Profiler Human Phospho-Kinase Array was performed at 6 h after ceritinib treatment to observe the signaling events prior to the initiation of apoptosis and caspase activation, when global protein degradation would have interfered with detection of signaling events. Analysis of the Proteome Profiler Human Phospho-Kinase Array revealed a decrease in phosphorylation levels of Akt (S473), Akt (T308), mTOR (S2448), PRAS40 (T246), and GSK-3α/β (S21/S9), indicating the suppression of the Akt signaling cascade (Table S3, Figure 4A,B, red arrows). Interestingly, a variable reduction in phosphorylation was observed for Fyn, FAK, Fgr, Lck, Lyn, Chk-2, and Yes, the kinases which are previously reported to be inhibited by ceritinib (Figure 4A,B) [28,29]. This observation showed that ceritinib can, indeed, inhibit multiple kinases, although the extent of inhibition may vary from in vitro observations (Figure S3A). Additionally, there was a decrease in phosphorylation at S392 of p53 (reduced from 1 to 0.64-fold) and an increase in phosphorylation of c-Jun at S63 (increased from 1 to 1.65-fold). Notably, the kinases involved in Akt pathways were the kinases most affected by the ceritinib treatment.

Western blot analysis confirmed that a ceritinib dose-dependently induced LC3-BII formation increased phosphorylation of p38 (Thr180/Tyr182) and decreased phosphorylation of Akt (Ser473), mTOR (Ser2448), and 4E-BP1 (Thr37/46), although p62/SQSTM1 expression did not significantly change at 6 h after the treatment (Figure 4C). Densitometry analysis of signaling proteins was shown in Figure S5.

### 2.7. Ceritinib and Cisplatin Combination Showed Synergistic Activity

**Figure 5:**
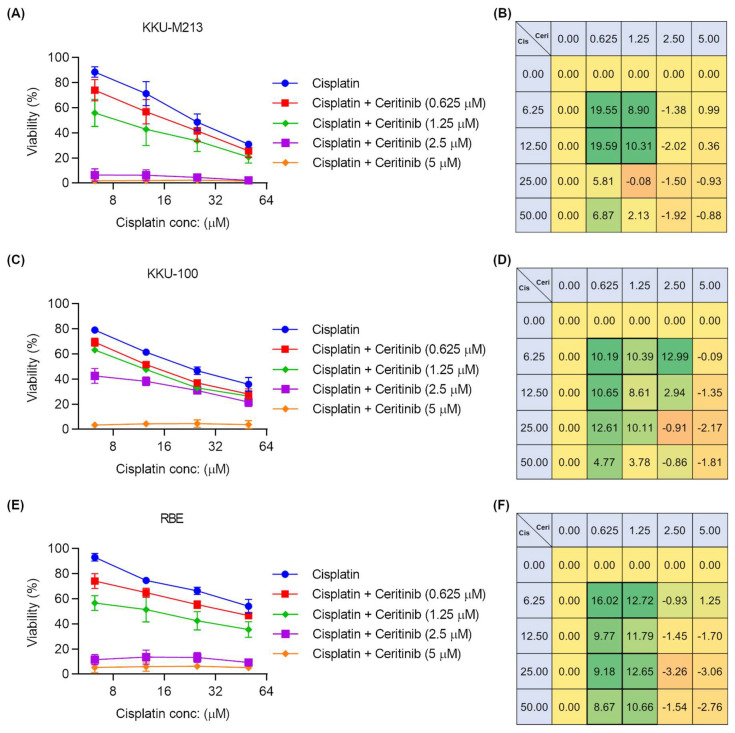
Ceritinib showed synergistic activities with cisplatin in CCA cells. (0, 0.625, 1.25, 2.5, and 5) μM concentrations of ceritinib and (0, 6.25, 12.5, 25, and 50) μM concentrations of cisplatin were applied as a treatment to KKU-M213, KKU-100, and RBE cells as 5 × 5 matrix combination. The cytotoxicity of ceritinib and cisplatin combinations and ZIP synergism scores of (**A**,**B**) KKU-M213, (**C**,**D**) KKU-100, and (**E**,**F**) RBE. The data are presented as means ± SEM of four independent experiments performed with duplicate wells. A stronger green color indicates the higher synergy score.

Cisplatin is a well-established chemotherapeutic agent in CCA treatment. Resistance to cisplatin has been reported to be associated with the induction of Akt expression in colon cancer [34], and Akt/mTOR pathway inhibition reduced cisplatin resistance in ovarian and lung cancer cells [35,36]. Here, we investigated the clinical potential of ceritinib, which is both cytotoxic and has Akt-pathway suppression activity in CCA, in combination with cisplatin using KKU-M213, KKU-100, and RBE cell lines. Ceritinib (0, 0.625, 1.25, 2.5, and 5 μM) and cisplatin (0, 6.25, 12.5, 25, and 50 μM) were applied as a treatment to CCA cells as a combination matrix for 72 h. The synergistic effect of the drug combination was evaluated using the ZIP synergy score, and calculated using SynergyFinder2.0 [37]. A ZIP synergy score of (−10 to 10) indicates an additive effect and (>10) indicates synergism. The relative cell viability inhibition ± SEM and zero interaction potency (ZIP) synergy score of each combination are provided in Table S4. Ceritinib showed synergistic activity with cisplatin at 0.625 and 1.25 µM, which is well within the plasma concentration range of ceritinib (1.4–2.3 μM) in KKU-M213, KKU-100, and RBE cell lines (Figure 5A–F). At the higher concentrations of ceritinib (2.5 and 5 μM), single drug treatment was killing more than 95% and synergism could not be accurately determined.

### 2.8. Ceritinib Showed Potent Cytotoxic Effects in Spheroid Models and Xenograft Models

**Figure 6:**
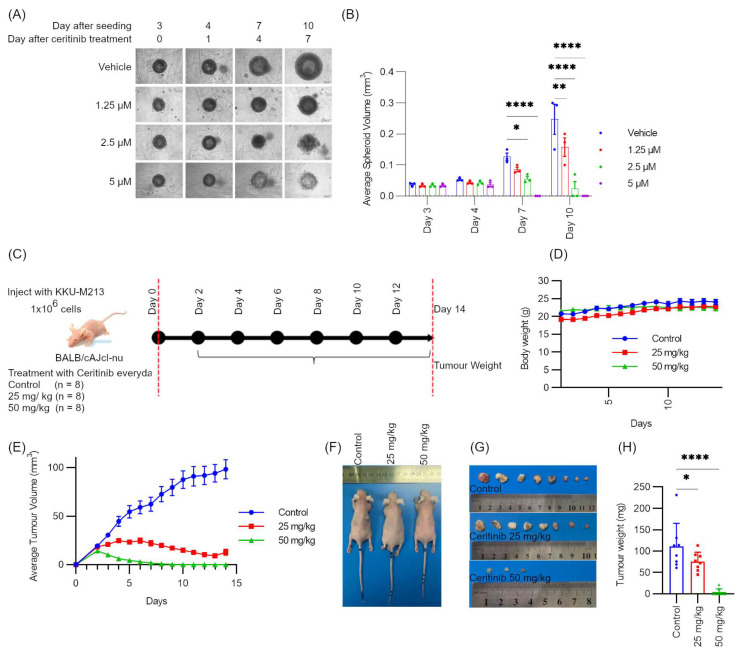
Anti-tumor activity of ceritinib in spheroid model and xenograft model systems. (**A**) Ceritinib significantly inhibited the growth of KKU-M213 spheroids. Ceritinib treatment was performed on day 3 after seeding the cells. (**B**) Spheroid volume analysis on day 3, 4, 7, and 10. Spheroid volume measurement was performed using cell-sense imaging software. The experiments were performed as three independent experiments. (**C**) Experiment outline of in vivo experiments. (**D**) Body weight (g) of mice during experiments. (**E**) Average tumor volume (mm^3^) measurement. Representative image of (**F**) mice on day 14, (**G**) tumors, and (**H**) tumor weight (mg). The data are presented as means ± SEM. * *p* < 0.005, ** *p* < 0.01 and **** *p* < 0.0001 compared to vehicle or control.

Since the potent cytotoxicity of ceritinib in a 2D culture system showed promising clinical applications, a monotypic 3D spheroid model was used to test the cytotoxicity of ceritinib. KKU-M213 spheroids were cultivated, as mentioned in Section 4. On the third day after seeding, the uniformity of spheroids was confirmed, and the spheroids were treated with ceritinib (1.25–5 μM). Ceritinib treatment significantly inhibited spheroid growth at a concentration of 1.25 μM after 7 days of treatment (*p* = 0.0013). Ceritinib at 2.5 and 5 μM completely eradicated the spheroids on day 7 and 4, respectively (Figure 6A,B).

To evaluate the therapeutic efficacy of ceritinib in xenograft models, BALB/cAJcl-nu mice were injected with 1 × 10^7^ KKU-M213A cells on day 0. The mice were divided into three groups: a vehicle control, a 25 mg/kg, and a 50 mg/kg treatment group, with each group consisting of eight mice (n = 8). Ceritinib administration began on day 1 and continued for a duration of 14 days. Throughout the study, no significant weight loss was observed in the animals (Figure 6C,D). Average tumor volume measurement showed that ceritinib suppressed the growth of tumors (Figure 6E,F). After sacrificing the animals on day 14, the tumors were excised and weighed. Ceritinib treatment resulted in a significant reduction in average tumor weight in both the 25 mg/kg group (*p* = 0.0285) and the 50 mg/kg group (*p* < 0.0001) (Figure 6G,H).

## 3. Discussion

CCA represents a significant clinical challenge, as surgical intervention remains the sole curative option. The standard chemotherapy regimens for CCA typically involve combinations of gemcitabine with cisplatin, oxaliplatin, capecitabine, or 5-FU [38,39]. To overcome the limitations of current treatment approaches, several clinical trials are investigating the efficacy of small molecule inhibitors as a stand-alone, or in combination with standard chemotherapies [40]. In our screening of receptor tyrosine kinase inhibitors on the KKU-M213 cell line, ceritinib emerged as the most cytotoxic compound among 112 RTK inhibitors, prompting further exploration of its therapeutic potential in CCA.

Our study showed ceritinib selectively targeted CCA cells at clinically relevant plasma concentrations, with differential sensitivity to immortalized cholangiocyte, MMNK-1, suggesting a potential sparing effect on normal cholangiocytes (Figure 1B,C). Intriguingly, CCA cells exhibited heightened sensitivity to ceritinib compared to non-CCA cells, despite their low level of or absence of ALK expression as evidenced by data mining in the CCLE and TCGA databases and silencing of ALK, respectively (Figure 1D–L). These findings indicate that ceritinib’s cytotoxic effects in CCA are independent of ALK expression or mutation status.

ALK mutation status in CCA is an extremely rare condition as reported by Nai-Jung Chiang in 2016, showing that ALK rearrangement in CCA is present in 1/110 cases [41]. Similarly, Jeremy Augustin and colleagues in 2020 found no ALK translocations or amplifications in a study involving 73 intrahepatic, 40 perihilar bile duct, and 36 distal extrahepatic CCAs [42]. This rarity of ALK mutations in CCA makes patient recruitment for clinical trials challenging. For instance, the clinical trial NCT02638909, which aimed to investigate the potential of ceritinib in patients with advanced gastrointestinal malignancies (including colorectal adenocarcinoma, cholangiocarcinoma, pancreatic adenocarcinoma, hepatocellular adenocarcinoma, gastric adenocarcinoma, and esophageal adenocarcinoma) harboring ALK and ROS1 rearrangements, and who lacked alternative therapeutic options, was terminated due to inadequate enrollment [43]. This lack of participation may be attributed to the rarity of ALK or ROS1 mutations in these cancers.

Upon further investigation into the mechanism of cell death, ceritinib was found to induce both apoptosis and autophagy in CCA cells as early as 6 h after treatment (Figure 2 and Figure 3). The Proteome Profiler Human Phospho-Kinase Array revealed that ceritinib treatment led to a reduction in the phosphorylation of various tyrosine kinases, including Fyn, FAK, Fgr, Lck, Lyn, Chk-2, and Yes1. Recently, Lin et al. demonstrated that the actual effectiveness of many anti-cancer drugs in ongoing clinical trials can be attributed to their off-target activities. On average, small molecule drugs were found to interact with a minimum of 6–11 distinct targets in addition to their intended pharmacological target [44,45,46]. Some RTKis, notably VEGFR-associated multi-targeted RTKis, exhibit a broader spectrum of targets beyond their primary focus. Despite their initial selectivity design, certain multi-targeted RTKis may inadvertently affect other unexpected targets [47,48]. The observations in KINOMEscan^®^ data and the Proteome Profiler Human Phospho-Kinase Array are in alignment with Lin’s report (Figure S4A and Figure 4A,B).

Additionally, a decrease in the phosphorylation of key signaling proteins within the PI3K/Akt/mTOR pathway, such as Akt (S473 and T308), mTOR (S2448), PRAS40 (T246), and GSK-3α/β (S21/S9), was observed (Figure 4A,B), in consistence with results from the pathway enrichment analysis, which showed that mTOR and MAPK signaling pathways were the key pathways affected by ceritinib (Figure S4C). However, the specific target kinase(s) responsible for Ceritinib’s cytotoxicity is not identified here.

The signaling changes after ceritinib treatment showed a dose-dependent decrease in phospho-Akt and an increase in phospho-p38, the signaling molecules which were reported to be involved in autophagy [49,50], and these changes were consistent with LC3-BII formation (Figure 4C and Figure S4C). Aberrant activation of the Akt pathway through overexpression, amplification, or constitutive phosphorylation is frequently observed in malignancies and contributes to tumor aggressiveness and drug resistance [51,52,53]. Similarly, CCA was reported to have increased PI3K/Akt activity [54], and the suppression of the Akt pathway was shown to decrease CCA cell viability [55,56]. 

The Akt pathway suppression by ceritinib in CCA led us to address the possibility of combining ceritinib with cisplatin, a common therapeutic drug in CCA. Notably, resistance to cisplatin has been reported to be associated with the induction of Akt expression in colon cancer [34], and Akt/mTOR pathway inhibition reduced cisplatin resistance in ovarian and lung cancer cells [35,36]. Our data showed that ceritinib exhibited synergistic activity with cisplatin at 0.625 and 1.25 µM (Figure 5), which are within the plasma concentration range of ceritinib in these cell lines, suggesting the combination of ceritinib and cisplatin is a feasible therapeutic strategy in CCA.

The 3D spheroid model provides a more realistic representation of the tumor microenvironment, encompassing crucial factors such as cellular heterogeneity and resistance to chemotherapy [57,58,59,60,61]. Ceritinib treatment effectively inhibited spheroid growth at a concentration of 1.25 μM, while higher concentrations led to complete spheroid eradication within a period of 4–7 days. In the xenograft model, treatment with ceritinib at a dosage of 50 mg/kg resulted in a significant reduction in tumor weight and volume across all the mice (Figure 6). This outcome aligns with a previous xenograft study utilizing the A375P melanoma cell line, which also lacked ALK rearrangements or mutations [62].

Our observations of ALK-independent cytotoxicity of ceritinib in CCA carries clinical significance, indicating ALK may not be a suitable marker for indication of ceritinib sensitivity in CCA. Similar findings were observed in a phase-I study where ceritinib, in combination with gemcitabine and cisplatin, conferred prolonged clinical benefits to a subset of evaluable CCA patients (n = 3/5) without ALK mutations [63].

However, in NSCLCs, ALK–tyrosine kinase inhibitors (TKIs) target oncogenic ALK-signaling and the tumors tends to get acquired resistance and relapse [64]. When the lung cancer cells evolved to be resistant to one type of ALK inhibitor, they showed collateral resistance to other ALK inhibitors (crizotinib, ceritinib, lorlatinib, and alectinib) [65]. Notably, in CCA cells, our findings showed that the cytotoxic mechanism of ceritinib was ALK-independent. Hence, how the resistance to ceritinib may develop in CCA would be an interesting area for future study. 

In summary, our study highlights the significant therapeutic promise of ceritinib in CCA, regardless of ALK expression or mutation status. Ceritinib targets critical survival pathways in CCA cells, at clinically relevant plasma concentrations, resulting in both apoptosis and autophagy induction. Initiation of clinical trials with ceritinib in CCA patients, irrespective of their ALK mutation status, with retrospective analysis on the clinical responses, is warranted to fully unlock ceritinib’s therapeutic potential in cholangiocarcinoma.

## 4. Materials and Methods

### 4.1. Cell Lines and Cell Cultures

Cell lines used for the studies are listed in Table 1. CCLP-1 cell line was cultured in DMEM, high glucose medium (Gibco, Life technologies, Grand Island, NY, USA) supplemented with 10% fetal bovine serum (FBS) (Gibco, Life technologies, Grand Island, NY, USA) and 1% MEM non-essential amino acids solution (Gibco, Life technologies, Grand Island, NY, USA). The rest of the cell lines were cultured in RPMI medium 1640 (Gibco, Life technologies, Grand Island, NY, USA) supplemented with 10% FBS. The cells were incubated at 37 °C in a humidified 5% CO_2_ atmosphere in the presence of 1× antibiotic-antimycotic (Gibco, Life technologies, Grand Island, NY, USA).

### 4.2. Chemical and Reagents

Ceritinib (HY-15656), NVP-TAE684 (HY-10192), ASP3026 (HY-13326), and bafilomycin A1 (HY-100558) were purchased from MedChemExpress, NJ, USA. GSK1904529A (S1093) was purchased from Selleckchem and FAK-inhibitor 14 (sc-203950) from Santa Cruz. RTK(i)s used for screening were purchased from MedChemExpress, NJ, USA and are listed in Table S1. The inhibitors drugs were prepared as 1–10 mM stock in DMSO according to the solubility. Poly (2-hydroxyethyl methacrylate) (PolyHEMA) (P3932-10G) was purchased from Sigma Aldrich, St. Louis, MO, USA. Corning^®^ Matrigel^®^ Basement Membrane Matrix, LDEV-free (354234) was purchased from Corning, Bedford, MA, USA.

### 4.3. Cell Viability Assays

Primary screening was performed by seeding the KKU-M213 cells (5000 cells/well) overnight, followed by treating with 10 μM RTK(i) as single well treatment, and cell viability was measured using resazurin reduction assay (HY-111391, MedChemExpress, NJ, USA) at 48. The viability was calculated as

$$\mathrm{Viability}\%=\frac{(\mathrm{Absorbance}\;\mathrm{of}\;\mathrm{Test}-\mathrm{Absorbance}\;\mathrm{of}\;\mathrm{Background}\;\left(100\%\;\mathrm{dead}\;\mathrm{cells}\right)}{(\mathrm{Absorbance}\;\mathrm{of}\;\mathrm{Vehicle}-\mathrm{Absorbance}\;\mathrm{of}\;\mathrm{Background}\;\left(100\%\;\mathrm{dead}\;\mathrm{cells}\right)}\times 100$$

The compounds with significant cytotoxicity of more than 90% cell death compared to positive control were validated in secondary screening as single set by treating with 0.01, 0.1, 1, and 10 μM and the viability was measured using MTT (3-(4,5-dimethylthiazol-2-yl)-2,5-diphenyl-2H-tetrazolium bromide) assay.

For cytotoxicity assays, the cells were seeded in a 96-well plate at density of 2500 cells per well overnight in 100 µL culture medium at 37 °C in a humidified 5% CO_2_ atmosphere. The next day, the cells were treated with 200 µL of varying concentrations of drugs. At 72 h after drug treatment, the drug containing medium was replaced with 150 µL of 0.5 mg/mL MTT (AppliChem, Darmstadt, Germany) prepared in 10% FBS supplemented RPMI medium from stock solutions and the cells were incubated for 3 h at 37 °C in a humidified 5% CO_2_ atmosphere. The medium was carefully removed so as not to disturb the MTT crystals and 200 µL of analytical grade DMSO (CAS:67-68-5, Fisher Chemical, Loughborough, UK) was added and absorbance at 540 nm was measured using a microplate reader (Multiscan EX, Thermo Labsystems, Helsinki, Finland) after shaking for 1 min. For ceritinib in combination with bafilomycin A1, the cells were treated with inhibitors for 1 h prior to ceritinib treatment in combination with the inhibitors. IC_50_s were calculated in GraphPad Prism 9. Otherwise, the detailed deviations were mentioned in text.

### 4.4. Colony Formation Assays

Cells were seeded in a 6-well plate at 1000 cells’ density overnight. The next day the cells were treated with varying concentrations of drug in 10% FBS supplemented medium (3 mL). The cells were grown for 10 days at 37 °C in a humidified 5% CO_2_ atmosphere. Then, the cells were washed with warm PBS one time before fixing with acetic acid/methanol (1:7 vol/vol) for 5 min. After fixing solution was removed, cells were washed with PBS one time, stained with 0.5% crystal violet (25% methanol) for 30 min, followed by washing with water.

### 4.5. Annexin V and PI Staining for FACS

CCA cells were seeded in a 6-well plate at 3 × 10^5^ density for 24 h. The next day, the old medium was removed and treated with 5 µM ceritinib or DMSO 0.001% as vehicle control for 24 h in 10% FBS supplemented medium. Then, the attached cells were trypsinized, combined with detached cells in the medium, and collected by spinning down, then were washed with PBS and resuspended in annexin V-binding buffer. Then, the cells were stained with annexin V (0.3 μg/mL) and PI (2 μg/mL) for 5 min at RT in the dark. FACS analysis was performed with FACScanto flow cytometer (Becton and Dickson, San Jose, CA, USA). The percentage of apoptotic cells is the addition of the percentage of early and late apoptotic cells.

### 4.6. Datamining of ALK Gene Expression and Mutation Status in CCA

We used FKPM (fragments per kilobase million) data from The Cancer Genome Atlas-Cholangiocarcinoma (TCGA-CHOL), which contains CCA (n = 36) and normal tissues (n = 9). The raw data of gene expression levels were log2 (FKPM + 1) transformed, and only the ALK gene expression was compared between CCA tumors and normal tissues [66]. Box plots to compare gene expression between tumor and normal were generated using GraphPad Prism 9. Statistical significance was determined using an unpaired *t*-test. Further, pre-processed and log transformed RNA seq data (TPM + 1) of CCA cell lines were obtained from publicly available data in Cancer Cell Lines Encyclopedia (CCLE) [27,67].

Ceritinib KINOMEscan—Dataset (ID:20329) (unpublished data) was obtained from Harvard Medical School LINCS (Library of Integrated Network-based Cellular Signatures) Center, which is funded by NIH grants U54 HG006097 and U54 HL127365. The kinases (n = 57) blocked by ceritinib down to 50% or lower in activity were chosen. The pathway enrichment analysis was performed using Enrichr [58,59].

### 4.7. siRNA-Mediated Gene Silencing

siRNA to ALK genes; siALK-1 5′-GAGUCUGGCAGUUGACUUCTT-3′ targeting Exon-1 of ALK mRNA [68] was synthesized by Eurofins Genomics, Ebensburg, Germany. KKU-M213 and HUCCT1 cell lines were seeded 200,000 cells per well in 6 well plates for 24 h before siRNA transfection. The cells were transfected with siRNA using Lipofectamine^®^ RNAiMAX reagent (Invitrogen, Life technologies, Carlsbad, CA, USA) following the manufacturer’s protocol for 24 h.

### 4.8. qRT-PCR

Total mRNA extraction was performed using Total RNA Mini Kit (Blood/Cultured cells) (Geneaid, New Taipei City, Taiwan) and reverse transcription was using ImProm-II™ Reverse Transcription System (Promega, Madison, WI, USA). qRT-PCR was performed using Faststart universal SYBR green Master (Roche, Mannheim, Germany). Specific primers to ALK gene (NM_004304.5) forward: 5′-GAGGGGGCGGCAAGATT-3′ and reverse: 5′-CTTGTGGCTCCTCCAAGCTC-3′ were used. 18S mRNA was employed as internal control gene using forward: 5′-CCATCCAATCGGTAGTAGCG-3′ and reverse: 5′-GTAACCCGTTGAACCCCATT-3′ primers. The gene expression was determined using the 2^−ΔΔCt^ technique, setting the vehicle as reference (100%).

### 4.9. SDS-PAGE and Immunoblotting

Total cellular protein was extracted using lysis buffer cocktail containing 1% (vol/vol) Triton-X (OmniPur, Calbiochem, Darmstadt, Germany), 150 mM NaCl, 50 mM Tris-HCl pH8.0, 1× protease inhibitor (cOmplete Mini EDTA-free, Roche, Mannheim, Germany), 50 mM NaF (Sigma-Aldrich, St. Louis, MO, USA), 40 mM β-glycerophosphate (Sigma-Aldrich, St. Louis, MO, USA), 2 mM sodium orthovanadate (Sigma-Aldrich, St. Louis, MO, USA), and 1 mM dithiothreitol (Amersham Biosciences, Uppsala, Sweden). Each protein sample of 20–40 µg was separated via SDS-PAGE (12% gel; 120 V for 2.5 h) followed by an electroblotting transfer of the protein to a nitrocellulose membrane at 30 V at 4 °C for 15 h. The membranes are cut into 2–3 pieces according to the molecular weights of the protein to be studied before blocking in 3% BSA at room temperature for 1 h and incubated with the primary antibodies at 4 °C overnight on a rocking platform. If 2 or more membranes were to be used, loading control protein was probed on each membrane and respective densitometry measurement of that loading control was used to analyze the expression changes. β-Actin was less variable than GAPDH among the cell lines and was used for comparing ALK expression among the cell lines. The next day, the membranes were washed with TBS with 0.1% Tween-20 (TBS/T) prior to incubation with horseradish peroxidase-conjugated secondary antibodies for 1 h at room temperature. Following 3 washes with TBS/T for 10 min, ECL Plus western blotting detection system (Bio-Rad Laboratories, Inc., Hercules, CA, USA) was used to visualize the immunoreactive bands in G:Box ChemiXL 1.4 (Syngene; Synoptics, Cambridge, UK). Antibodies and the dilutions were mentioned in Table 2. Densitometry analysis was performed using ImageJ 1.54d [69].

### 4.10. Immuno-Fluorescence and Fluorescent Microscopy

The cells were seeded at 50,000 cells per well in 8 well chambered slides (Lab-Tek, Rochester, NY, USA). For LC3-B detection, cells were treated with ceritinib for 6 h, washed with warm serum-free medium and fixed with 4% paraformaldehyde + 2% sucrose/PBS for 20 min. The fixed cells were washed with PBS for 10 min 3 times and blocked with 1% BSA/PBS for 1 h and then probed with primary antibodies (1:200/1% BSA/PBS) overnight. The next day, primary antibody solution was removed and washed with PBS 10 min 3 times and probed with anti-rabbit secondary antibodies conjugated with Alexa488 (A11034, Thermo Fisher, Eugene, OR, USA). Then, the cells were counter-stained with DAPI (Thermo Fisher Scientific, Waltham, MA, USA). Fluorescent image acquisition was performed using FV10i confocal laser scanning microscope (Olympus, Tokyo, Japan).

For DAPI staining for nuclear morphology, the cells were seeded at 10,000 cells per well in 8 well chambered slides overnight. The next day, the cells were treated with ceritinib for 24 h, washed with warm serum-free medium and fixed with 4% paraformaldehyde + 2% Sucrose/PBS for 20 min. The fixed cells were washed with PBS for 10 min 3 times and stained with DAPI (Thermo Fisher Scientific, Waltham, MA, USA) for 45 min, followed by washing with PBS 10 min 3 times. Fluorescent image acquisition was performed by excitation with UV using BX53 microscope (Olympus, Tokyo, Japan).

### 4.11. Autophagy Flux Study

GFP-LC3-RFP expressing plasmids were a kind gift from Asst. Prof. Marisa Ponpuak, Department of Microbiology, Faculty of Science, Mahidol University, Thailand. CCA cells were seeded at density of 500,000 cells per well in 60 mm dish and transfected with 1 µg of GFP-LC3-RFP plasmids for 24 h. The next day, the cells were trypsinized and seeded into 8 well chambered slides at 50,000 cells per well density (Lab-Tek, Rochester, NY, USA) and cultured for 24 h. The next day, the cells were treated with ceritinib for 6 h. After washing with warm serum-free medium, the cells were fixed 4% paraformaldehyde + 2% sucrose/PBS for 20 min. Then, the cells were counter-stained with DAPI. Fluorescent image acquisition was performed using FV10i confocal laser scanning microscope (Olympus, Tokyo, Japan).

### 4.12. Antibody Array

Proteome Profiler^TM^ Array (Human Phospho-Kinase Array Kit, ARY003B) was purchased from R&D Systems, Ninneapolis, MN, USA. KKU-M213 cells were seeded at 800,000 cells per plate in 10 cm cell culture plates overnight. The next day, the cells were treated with 5 μM ceritinib for 6 h and the protein extraction was performed following the manufacturer’s protocol. The membranes were probed with 400 μg total protein extract and the immunoreactive spots were visualized in G:Box ChemiXL 1.4 (Syngene; Synoptics, Cambridge, UK). Densitometry of array spots was analyzed by Image Lab Software 6.1 (Bio-Rad).

### 4.13. Drug Synergism Study

KKU-M213, KKU-100, and RBE cells were seeded in a 96-well plate at 2500 per well density overnight. The next day, the cells were treated with 5 × 5 matrix combination of ceritinib (0, 0.625, 1.25, 2.5, and 5 μM) and ciplatin (0, 0.625, 1.25, 2.5, and 5 μM) concentrations in final volume of 200 μL for 72 h before MTT assay. ZIP synergy score was calculated using SynergyFinder2.0 [37].

### 4.14. Spheroid Culture

A 120 mg/mL stock solution of polyHEMA was prepared in 95% ethanol (10×). 1× solution was prepared by dilution with 95% ethanol. Corning (REF 3788, New York, NY, USA) clear round bottom plates were coated with 60 uL of 1.2 mg/mL polyHEMA and the ethanol was allowed to evaporate overnight. The coated plates were stored at 4 °C until use (less than one month). A total of 70–90% confluent KKU-M213 cells were trypsinized in 1× Tryspin-EDTA (Gibco) × 5 min, blocked with complete medium, and centrifuged, counted, and resuspended into 5000 cells/mL suspension in 10% FBS supplemented DMEM-high glucose medium. Matrigel^®^ was pre-thawed on ice for 1–2 h and mixed into the cell suspension to make 2.5% concentration. The mixture of cells and Matrigel (200 μL) was seeded in polyHEMA coated 96 well round-bottom plates, using ice-cold tips to avoid Matrigel solidification (1000 cells per well). The microplates were centrifuged at 500× *g* for 5 min at 4 °C to help cell aggregation. The process of mixing cell into cold medium to centrifugation was carried out within 10–15 min to reduce cold stress and cell death [70].

The plates were incubated at 37 °C in a humidified 5% CO_2_ atmosphere and spheroid formation was observed daily and recorded at day 3, 4, 7, and 10 using live cell imaging microscopy (IX83, Olympus, Tokyo, Japan) after initial observations to optimize the time points to record. Spheroid volume (V) was calculated according to the formula V = [(a^2^) × (b)]/2, in which a and b represent the minor and major diameter, respectively. Ceritinib was applied as a treatment to the spheroids on day 3 after confirmation of spheroid formation.

### 4.15. Xenograft Model

BALB/cAJcl-nu mice (male and 6–8 weeks of age) were purchased from the Nomura Siam International co. Ltd., Bangkok, Thailand. Ethical approval for animal experimental protocol was approved by Naresuan University Animal Care and Use Committee (NU-AE640705).

#### 4.15.1. Preparing Cells for Transplantation

KKU-213A cells were maintained in complete medium at 37 °C in 5% CO_2_. After pelleting the cells at 60× *g* for 5 min, the supernatant was removed, and cells were resuspended homogeneously in PBS. Then, the cell concentration was measured and adjusted to 1 × 10^7^ cells/mL.

#### 4.15.2. Subcutaneous Injection of Cells and Treatment

An amount of 1 × 10^6^ KKU-213A cells were subcutaneously injected into the right flank region of BALB/cAJcl-nu mice. On the next day (Day 1), mice were randomized into three groups (n = 8): a control group treated with vehicle alone (0.5% *w*/*w* methylcellulose and 0.5% *w*/*w* Tween 80), an experimental group treated with 25 mg/kg ceritinib, or an experimental group treated with 50 mg/kg ceritinib administered by oral gavage every day for 14 days. Tumor growth was monitored using a vernier caliper every day and the tumor volume was calculated using the formula: tumor volume = 1/2 (length × width^2^). Body weight was also recorded to observe the mice’s condition. On day 14, the mice were sacrificed by anesthetizing with i.p. thiopental (100 mg/kg), and tumors were removed and weighed.

### 4.16. Statistical Analysis

Data presented are the mean ± standard error of the mean (SEM) of three independent experiments, otherwise indicated. Comparisons of data between groups were carried out with Student’s *t*-test, one-way ANOVA, or two-way ANOVA as mentioned in the figure legends. An associated probability (*p*) of <0.5% was considered significant.

## Figures and Tables

**Table 1:** List of cell lines used and source.

Cell-Line	Cell Type	Identifier
MMNK-1	Immortalized Cholangiocytes	JCRB1554
HuCCA-1	Cholangiocarcinoma	JCRB1657
KKU-M055	Cholangiocarcinoma	JCRB1551
KKU-100	Cholangiocarcinoma	JCRB1568
KKU-M213	Cholangiocarcinoma	JCRB1557
RBE	Cholangiocarcinoma	RCB1292
TFK-1	Cholangiocarcinoma	RCB2537
HuCCT1	Cholangiocarcinoma	JCRB0425
CCLP1	Cholangiocarcinoma	Cellosaurus RRID: CVCL_0205
HepG2	Liver Cancer	ATCC HP-8065
A549	Non-small cell lung cancer	ATCC CCL-185
H2228	Lung Cancer	ATCC CRL-5935
H1299	Lung Cancer	ATCC CRL-5803
PC3	Prostate Cancer	ATCC CRL-1435

**Table 2:** List of antibodies.

Antibody	Type	Organism	Company	Dilution
ALK (D5F3) 3633T	Monoclonal	Rabbit	Cell Signaling Technology^®^, Beverly, MA, USA	1:1000
Phospho-ALK (Tyr1604) #3341	Monoclonal	Rabbit	Cell Signaling Technology^®^, Beverly, MA, USA	1:1000
Phospho-Akt (Ser473) (193H12) #4058	Monoclonal	Rabbit	Cell Signaling Technology^®^, Beverly, MA, USA	1:1000
Total Akt (BDI111) sc-56878	Monoclonal	Mouse	Santa Cruz Biotechnology, Inc., Santa Cruz, CA, USA	1:1000
Phospho-p38 MAPK (Thr180/Tyr182) #9211	Monoclonal	Rabbit	Cell Signaling Technology^®^, Beverly, MA, USA	1:1000
p38 MAPK #9212	Monoclonal	Mouse	Cell Signaling Technology^®^, Beverly, MA, USA	1:1000
GAPDH (0411) sc-47724	Monoclonal	Mouse	Santa Cruz Biotechnology, Inc., Santa Cruz, CA, USA	1:2000
β-actin A1978	Monoclonal	Mouse	Sigma Aldrich	1:10,000
PARP #9542	Monoclonal	Rabbit	Cell Signaling Technology^®^, Beverly, MA, USA	1:1000
Cleaved PARP (Asp214) (D64E10) XP^®^ #5625	Monoclonal	Rabbit	Cell Signaling Technology^®^, Beverly, MA, USA	1:1000
Caspase-3 Antibody #9662	Monoclonal	Rabbit	Cell Signaling Technology^®^, Beverly, MA, USA	1:1000
Cleaved Caspase-3 (Asp175) (5A1E) #9664	Monoclonal	Rabbit	Cell Signaling Technology^®^, Beverly, MA, USA	1:1000
LC3B #2775	Monoclonal	Rabbit	Cell Signaling Technology^®^, Beverly, MA, USA	1:1000
Anti-SQSTM1/p62 ab56416	Monoclonal	Mouse	Abcam, Cambridge, MA, USA	1:1000
Phospho-mTOR (Ser2448) (D9C2) XP^®^ #5536	Monoclonal	Rabbit	Cell Signaling Technology^®^, Beverly, MA, USA	1:1000
Phospho-4E-BP1 (Thr37/46) (236B4) #2855	Monoclonal	Rabbit	Cell Signaling Technology^®^, Beverly, MA, USA	1:1000
Anti-rabbit IgG, HRP-linked Antibody #7074	Polyclonal	-	Cell Signaling Technology^®^, Beverly, MA, USA	1:1000
Anti-mouse IgG, HRP-linked Antibody #7076	Polyclonal	-	Cell Signaling Technology^®^, Beverly, MA, USA	1:1000

## Data Availability

The original contributions presented in the study are included in the article/Supplementary Material. Further inquiries can be directed to the corresponding author(s).

## Supplementary Materials

The following supporting information can be downloaded at: https://www.mdpi.com/article/10.3390/ph17020197/s1, Figure S1: Secondary screening of 29 RTKis in KKUM213 cells; Figure S2: Inhibition of ALK phosphorylation by Ceritinib; Figure S3: Ceritinib is more cytotoxic to CCA cells than other ALKi(s); Figure S4: Signaling pathways predicted to be altered by Ceritinib treatment in CCA; Figure S5: Densitometry analysis of Figure 4C; Table S1: Primary screening of tyrosine kinase inhibitors; Table S2: Secondary screening of tyrosine kinase inhibitors; Table S3: Antibody array analysis with respective co-ordinates; Table S4: ZIP synergy scores ceritinib and cisplatin combination in KKU-M213, KKU-100 and RBE cells.
