# Supplementary material for: Therapeutic Implications of Ceritinib in Cholangiocarcinoma beyond ALK Expression and Mutation
Source: Pharmaceuticals (Basel). 2024 Feb 2;17(2):197. doi: 10.3390/ph17020197 (PMC10892566; doi:10.3390/ph17020197)
Supplement: Supplementary file 1 [file pharmaceuticals-17-00197-s001.zip › Supplementary Figures.pdf]

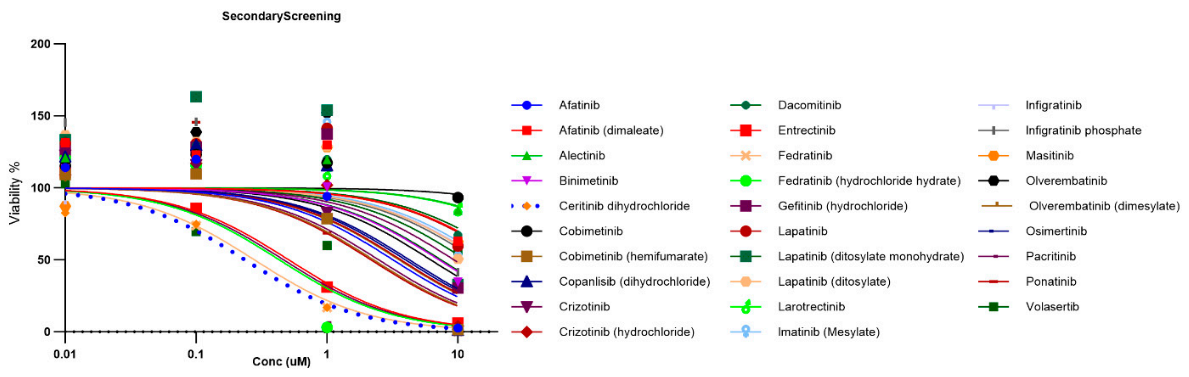

Figure S1. Secondary screening of 29 RTKis in KKUM213 cells. 0.01 -10 µM of drugs are treated to KKU-M213 cells for 48 hours before MTT assay. Ceritinib dihydrochloride (blue dotted line) is the most sensitive compound to KKU-M213 cells.

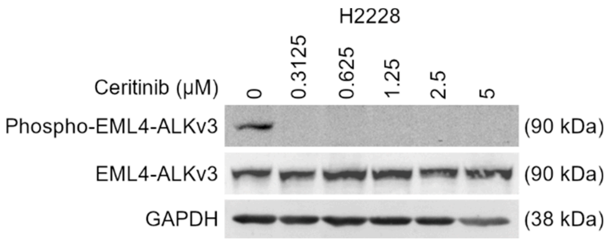

Figure S2. Inhibition of ALK phosphorylation by Ceritinib. H2228 (ALK-fusion positive cell line) cells were seeded in 6 well plate and treated with various concentration of ceritinib (0-5 µM) for 24 h at 80% confluence and total protein extraction was performed for WB analysis. GAPDH was used as loading control.

(A)

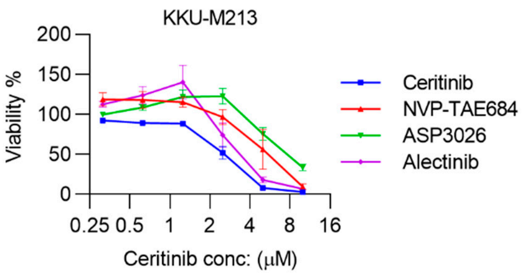

(B)

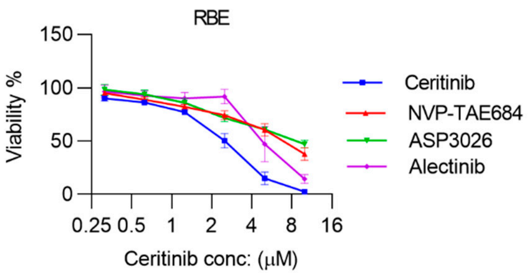

(C)

	KKU-M213		RBE	
	IC_{50}	SEM	IC_{50}	SEM
Ceritinib	2.49	0.13	2.29	0.15
NVP-TAE684	5.37	0.70	6.79	0.72
ASP3026	7.93	0.95	8.18	0.93
Alectinib	3.39	0.60	4.96	0.49

(D)

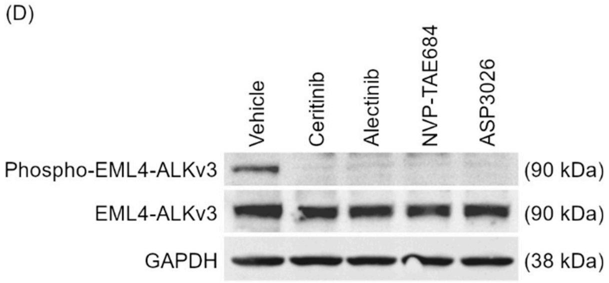

Figure S3. Ceritinib is more cytotoxic to CCA cells than other ALKi(s). Viability of (A) KKU-M213 and (B) RBE cells line after treatment with Ceritinib, NVP-TAE684, ASP3036 or Alectinib for 72 hours. (C) IC50s of ALKi(s) in KKU-M213 and RBE cells. (D) ALK phosphorylation inhibitory action of ALKi(s) in H2228 as positive control cells.

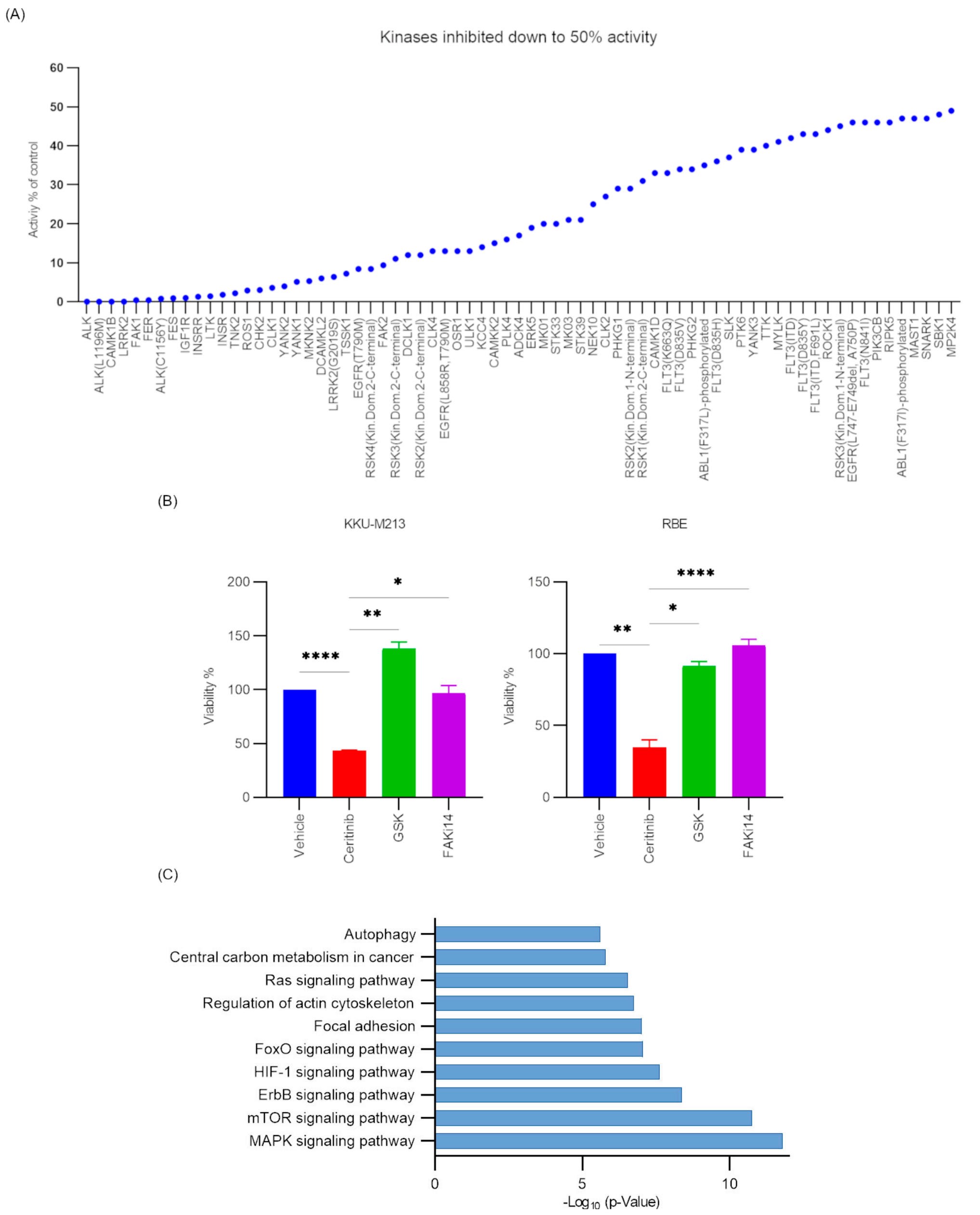

Figure S4. Signaling pathways predicted to be altered by Ceritinib treatment in CCA. **(A)** Kinases inhibited by ceritinib up to 50% at 1 μM concentration (cell free KINOMEscan) **(B)** CCA cell viability % after ceritinib, GSK1904529A and FAK inhibitor 14 treatment for 24 h in KKU-M213 and RBE cells **(C)** Gene enrichment analysis (KEGG pathway) of 57 kinases inhibited by ceritinib. *$p < 0.005$, **$p < 0.01$, ***$p < 0.001$, ****$p<0.0001$ compared to vehicle.

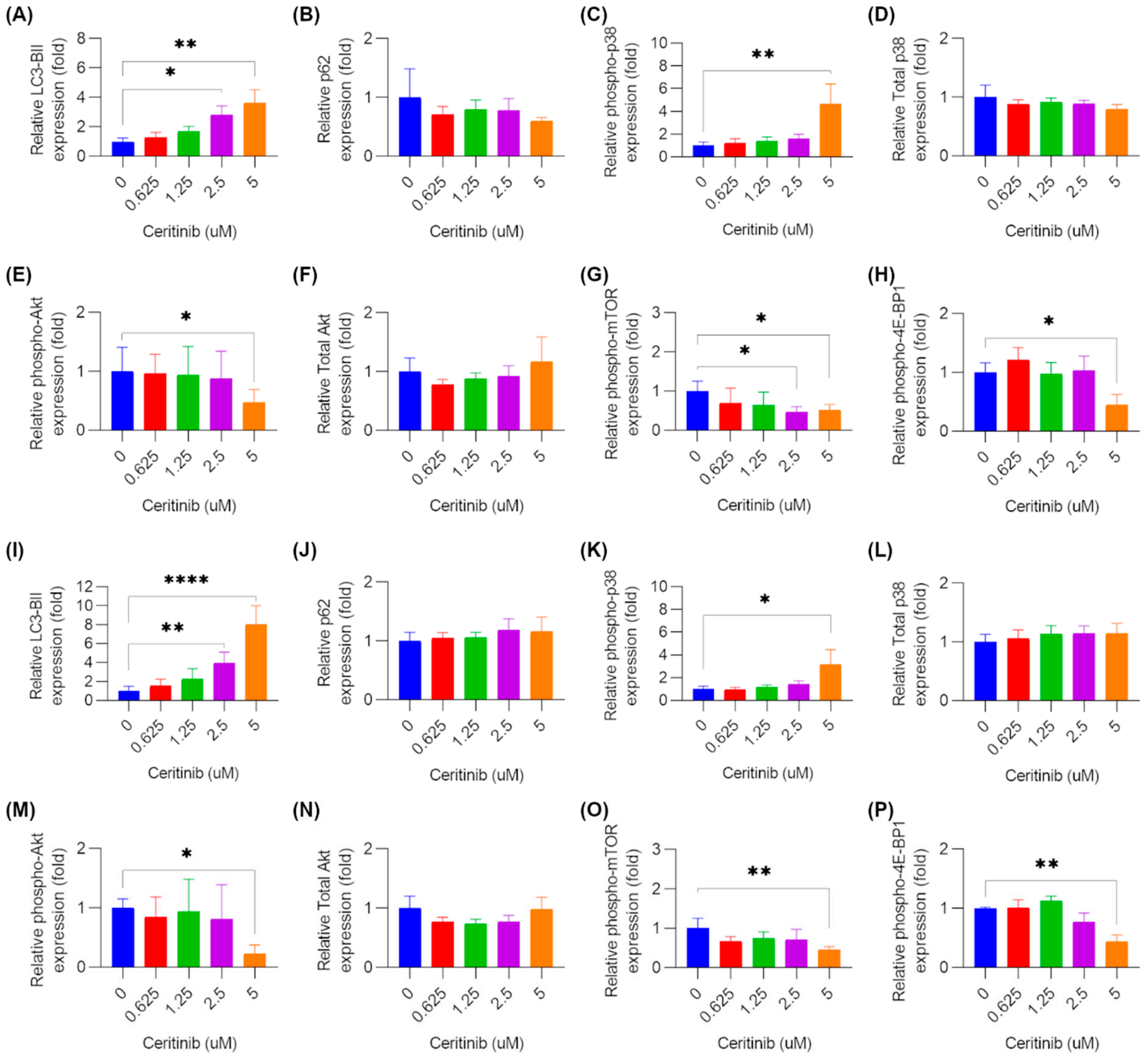

Figure S5. Densitometry analysis of Fig. 4C. Densitometry measurement was performed by ImageJ. Protein/GAPDH ratio for each experiment was determined. The average of ratio was calculated, and vehicle sample was referred as 1. The data are presented as means ± SEM of four independent experiments. *p < 0.005, **p < 0.01, ***p < 0.001, ****p<0.0001 compared to vehicle.
