# Supplementary material for: Therapeutic Implications of Ceritinib in Cholangiocarcinoma beyond ALK Expression and Mutation
Source: Pharmaceuticals (Basel). 2024 Feb 2;17(2):197. doi: 10.3390/ph17020197 (PMC10892566; doi:10.3390/ph17020197)
Supplement: Supplementary file 1 [file pharmaceuticals-17-00197-s001.zip › Supplementary Tables.pdf]

Table S1 Primary screening of tyrosine kinase inhibitors.

Name	Viablity % (10 µM)	Name	Viablity % (10 µM)
Volasertib	-103.10	Neratinib	32.22
Afatinib	-43.63	Sorafenib	33.56
Entrectinib	-42.60	Ceritinib	35.16
Fedratinib (hydrochloride hydrate)	-38.08	Asciminib (hydrochloride)	36.35
Cobimetinib	-30.39	Sunitinib	37.15
Binimetinib	-25.86	Gilteritinib	37.34
Cobimetinib (hemifumarate)	-21.13	Brigatinib	39.57
Copanlisib (dihydrochloride)	-18.68	Icotinib	41.44
Filgotinib	-17.72	Lonafarnib	45.28
Ponatinib	-12.81	Masitinib (mesylate)	46.18
Masitinib	-11.87	Sorafenib (Tosylate)	48.87
Crizotinib	-8.03	Trametinib	49.69
Lapatinib (ditosylate monohydrate)	-7.83	Lazertinib	50.91
Imatinib (Mesylate)	-6.92	Nilotinib	51.45
Dacomitinib	-6.34	Regorafenib (monohydrate)	51.59
Olverembatinib (dimesylate)	-5.93	Trametinib (DMSO solvate)	57.25
Fedratinib	-5.38	Gefitinib	58.04
Osimertinib	-4.36	Nilotinib (monohydrochloride monohydrate)	59.11
Infigratinib	-3.67	Erlotinib (Hydrochloride)	59.80
Crizotinib (hydrochloride)	-2.91	Tucatinib (hemiethanolate)	61.84
Pacritinib	-1.66	Lenvatinib	63.19
Lapatinib (ditosylate)	0.45	Nintedanib esylate	64.58
Gilteritinib hemifumarate	0.74	Flumatinib	64.88
Gefitinib (hydrochloride)	2.01	Nintedanib	66.13
Afatinib (dimaleate)	2.19	Imatinib	68.57
Ceritinib dihydrochloride	3.00	Ibrutinib	69.46
Infigratinib phosphate	3.12	Lorlatinib	70.48
Bortezomib	4.95	Toceranib	70.96
Alectinib	6.33	Erlotinib	71.72
Olverembatinib	6.35	Asciminib	74.70
Toceranib (phosphate)	6.87	Baricitinib (phosphate)	77.61
Lapatinib	7.29	Flumatinib (mesylate)	78.47
Fostamatinib Disodium	9.66	Icotinib (Hydrochloride)	78.48
Osimertinib (mesylate)	10.69	Ibrutinib (Racemate)	82.00
Olmutinib	11.41	Acalabrutinib	83.86
Almonertinib	12.10	Tirabrulinib	84.27
Sulfatinib	13.29	Tucatinib	84.71
Dinaciclib	14.54	Zanubrutinib	85.99
Almonertinib (mesylate)	15.34	Upadacitinib	86.64

Pemigatinib	17.40	Lenvatinib (mesylate)	90.32
Futibatinib	18.46	Ruxolitinib	90.71
Almonertinib (hydrochloride)	18.51	Tirabrutinib (hydrochloride)	92.67
Mobocertinib (succinate)	19.88	Abrocitinib	93.84
Selumetinib	21.71	Ruxolitinib (S enantiomer)	94.64
Fostamatinib (disodium hexahydrate)	21.82	Pexidartinib	94.94
Sunitinib (Malate)	22.48	Ruxolitinib (phosphate)	95.32
Regorafenib	24.57	Sirnotinib	96.56
Aleclinib (Hydrochloride)	24.71	Pexidartinib (hydrochloride)	98.10
Erdafitinib	25.41	Oclacitinib (maleate)	98.76
Fostamatinib	26.41	Orelabrutinib	100.12
Bosutinib	28.36	Savolitinib	101.81
Pazopanib	30.40	Baricitinib	102.62
Avapritinib	30.81	Quizartinib	104.22
Selumetinib (sulfate)	30.81	Capmatinib	104.47
Vandetanib	31.54	Tofacitinib	108.11
Peficitinib	31.85	Osimertinib (dimesylate)	548.15

Table S2 Secondary screening of tyrosine kinase inhibitors.

	Concentrations				
Name	0.01 uM	0.1 uM	1 uM	10 uM	IC50
Ceritinib dihydrochloride	82.6332	74.66701	16.90574	-0.12807	0.236473
Fedratinib	122.9252	73.71926	16.85451	0.256148	0.278693
Fedratinib (hydrochloride hydrate)	120.702	115.7593	3.044413	2.256447	0.431779
Pacritinib	110.7229	119.1161	6.47482	1.370332	0.466223
Entrectinib	110.7268	85.7049	31.1296	6.063923	0.509367
Ponatinib	132.0917	145.5946	68.62464	2.005731	2.164192
Cobimetinib (hemifumarate)	109.1944	109.7636	78.52452	1.641856	2.269627
Crizotinib	122.7715	116.0348	82.94057	-0.33299	2.482087
Afatinib	114.12	119.8774	94.04553	2.539405	3.191171
Olverembatinib (dimesylate)	104.2902	106.3634	103.628	2.159516	3.55028
Crizotinib (hydrochloride)	109.1332	117.0845	101.8983	4.118911	3.669138
Osimertinib	119.877	119.6721	113.2684	-0.53791	3.976552
Copanlisib (dihydrochloride)	121.5849	129.5096	115.4116	1.07268	4.254929
Olverembatinib	115.1594	138.6792	152.2121	1.7892	6.185381
Binimetinib	113.45	121.6495	100.5754	34.64397	7.151511
Volasertib	102.6708	69.57093	59.9387	56.19527	7.415706
Gefitinib (hydrochloride)	125.6584	128.2435	137.352	30.36965	9.312106
Lapatinib (ditosylate monohydrate)	133.3897	163.2278	153.9261	32.85818	11.50228
Infigratinib phosphate	145.3491	145.9773	153.2979	41.41097	13.96846
Lapatinib (ditosylate)	136.5194	131.8602	128.9483	50.59952	15.0874
Infigratinib	91.41906	99.43648	82.68443	63.49898	15.50367
Imatinib (Mesylate)	123.2665	123.9188	143.8753	51.2201	17.02592
Lapatinib	130.8888	130.5823	141.3748	59.58844	22.3279
Masitinib	87.27325	126.1733	128.2177	62.59718	22.58893
Afatinib (dimaleate)	131.3754	122.9943	129.9785	62.9298	23.20926
Dacomitinib	119.0455	129.5534	119.9869	67.18476	25.75415
Larotrectinib	109.8538	121.8892	110.0935	85.03956	65.95993
Alectinib	120.9016	123.207	119.5184	84.34939	69.04756
Cobimetinib	115.4772	123.1173	117.4037	93.19177	207.8059

Table S3 Antibody array analysis with respective co-ordinates.

Coordinate	Target/Control	Phosphorylation Site	Vehicle	Ceritinib	Fold Change
A-A1, A2	Reference Spot	___			
A-A3, A4	p38α	T180/Y182	1792.5	1425	0.79
A-A5, A6	ERK1/2	T202/Y204, T185/ Y187	7134.5	6173	0.87
A-A7, A8	JNK 1/2/3	T183/Y185, T221/ Y223	5505	4388	0.80
A-A9, A10	GSK-3α/β	S21/S9	10039.5	6787.5	0.68
B-A13, A14	p53	S392	1321	847	0.64
B-A17, A18	Reference Spot	___			
A-B3, B4	EGF R	Y1086	2746	2278.5	0.83
A-B5, B6	MSK1/2	S376/S360	1864.5	1529.5	0.82
A-B7, B8	AMPKα1	T183	2775.5	3469	1.25
A-B9, B10	Akt 1/2/3	S473	4891.5	3212.5	0.66
B-B11, B12	Akt 1/2/3	T308	1140	1081	0.95
B-B13, B14	p53	S46	1376	1174.5	0.85
A-C1, C2	TOR	S2448	1398.5	1089	0.78
A-C3, C4	CREB	S133	2300.5	1941.5	0.84
A-C5, C6	HSP27	S78/S82	1398	1053.5	0.75
A-C7, C8	AMPKα2	T172	2217.5	1781	0.80
A-C9, C10	β-Catenin	___	1656	1156.5	0.70
B-C11, C12	p70 S6 Kinase	T389	864.5	802	0.93
B-C13, C14	p53	S15	513	510	0.99
B-C15, C16	c-Jun	S63	5203.5	8592	1.65
A-D1, D2	Src	Y419	3072	2700.5	0.88
A-D3, D4	Lyn	Y397	1277.5	983	0.77
A-D5, D6	Lck	Y394	914.5	693	0.76
A-D7, D8	STAT2	Y689	2375.5	1710.5	0.72
A-D9, D10	STAT5a	Y694	821.5	664.5	0.81
B-D11, D12	p70 S6 Kinase	T421/S424	978	937.5	0.96
B-D13, D14	RSK1/2/3	S380/S386/S377	696	639.5	0.92
B-D15, D16	eNOS	S1177	651	665	1.02

A-E1, E2	Fyn	Y420	1148.5	753	0.66
A-E3, E4	Yes	Y426	1887	1544	0.82
A-E5, E6	Fgr	Y412	824.5	578.5	0.70
A-E7, E8	STAT6	Y641	1823.5	1340	0.73
A-E9, E10	STAT5b	Y699	1062.5	901.5	0.85
B-E11, E12	STAT3	Y705	948	904	0.95
B-E13, E14	p27	T198	669.5	722	1.08
B-E15, E16	PLC-γ1	Y783	1176	1148	0.98
A-F1, F2	Hck	Y411	1655	1158.5	0.70
A-F3, F4	Chk-2	T68	2213	1722.5	0.78
A-F5, F6	FAK	Y397	4383	3055.5	0.70
A-F7, F8	PDGF Rβ	Y751	1294	1001.5	0.77
A-F9, F10	STAT5a/b	Y694/Y699	1751	1327.5	0.76
B-F11, F12	STAT3	S727	1594	1437	0.90
B-F13, F14	WNK1	T60	2566	2391	0.93
B-F15, F16	PYK2	Y402	1162	1129.5	0.97
A-G1, G2	Reference Spot	___			
A-G3, G4	PRAS40	T246	6143	3693	0.60
A-G9, G10	PBS (Negative Control)	___			
B-G11, G12	HSP60	___	4033.5	3768	0.93
B-G17, G18	PBS (Negative Control)	___			

Table S4 ZIP synergy scores ceritinib and cisplatin combination in KKU-M213, KKU-100 and RBE cells.

			KKU-M213				KKU-100				RBE			
Ceri	Cis	Unit	Relative Inhibition			ZIP	Relative Inhibition			ZIP	Relative Inhibition			ZIP
0	0	µM	0.00	±	0.00	0.00	0.00	±	0.00	0.00	0.00	±	0.00	0.00
0.62	0	µM	8.15	±	4.66	0.00	18.17	±	2.19	0.00	15.17	±	9.41	0.00
1.25	0	µM	42.93	±	9.40	0.00	25.51	±	3.95	0.00	35.90	±	7.86	0.00
2.5	0	µM	97.74	±	4.03	0.00	48.69	±	8.18	0.00	89.47	±	6.44	0.00
5	0	µM	98.52	±	0.56	0.00	96.77	±	1.82	0.00	95.96	±	2.08	0.00
0	6.25	µM	8.75	±	4.18	0.00	19.95	±	2.15	0.00	7.73	±	3.00	0.00
0.62	6.25	µM	25.39	±	8.39	19.55	30.04	±	3.29	10.19	28.99	±	5.88	16.02
1.25	6.25	µM	48.19	±	10.80	8.90	37.56	±	2.05	10.39	43.38	±	5.97	12.72
2.5	6.25	µM	98.13	±	4.94	-1.38	62.22	±	6.00	12.99	89.92	±	4.11	-0.93
5	6.25	µM	98.41	±	0.84	0.99	96.96	±	1.40	-0.09	96.46	±	1.97	1.25
0	12.5	µM	23.35	±	9.61	0.00	39.13	±	2.00	0.00	25.30	±	1.55	0.00
0.62	12.5	µM	46.72	±	9.69	19.59	49.48	±	1.96	10.65	36.25	±	3.57	9.77
1.25	12.5	µM	64.24	±	13.01	10.31	51.94	±	1.12	8.61	56.35	±	9.92	11.79
2.5	12.5	µM	97.68	±	4.25	-2.02	64.43	±	3.38	2.94	89.92	±	5.54	-1.45
5	12.5	µM	98.04	±	0.68	0.36	96.15	±	1.39	-1.35	94.98	±	1.83	-1.70
0	25	µM	46.89	±	6.56	0.00	51.47	±	3.16	0.00	32.52	±	2.97	0.00
0.62	25	µM	51.07	±	8.40	5.81	63.14	±	1.94	12.61	46.08	±	2.99	9.18
1.25	25	µM	65.81	±	8.42	-0.08	67.01	±	1.47	10.11	63.49	±	7.28	12.65
2.5	25	µM	97.43	±	2.25	-1.50	69.31	±	1.74	-0.91	88.79	±	3.81	-3.26
5	25	µM	97.92	±	0.70	-0.93	96.33	±	1.50	-2.17	94.24	±	1.35	-3.06
0	50	µM	70.18	±	2.54	0.00	66.01	±	5.41	0.00	44.47	±	5.41	0.00
0.62	50	µM	77.43	±	5.31	6.87	71.78	±	4.12	4.77	54.15	±	2.62	8.67
1.25	50	µM	83.14	±	4.85	2.13	72.66	±	2.07	3.78	70.23	±	6.20	10.66
2.5	50	µM	98.11	±	0.65	-1.92	78.20	±	3.54	-0.86	91.80	±	2.61	-1.54
5	50	µM	98.44	±	0.55	-0.88	97.15	±	1.62	-1.81	95.40	±	1.26	-2.76
